# Real-world clinical outcomes of Trastuzumab Deruxtecan in HER2-expressing ovarian and endometrial Cancer

**DOI:** 10.1016/j.gore.2026.102183

**Published:** 2026-08-12

**Authors:** Jordyn Silverstein, Oladunni Alomaja, Deandra Chatram, Maryam Hajiabbasi, Eliya Shachar, Chi-Hong Tseng, Shivani Thaker, Aditya Bardia, Beth Karlan, Andrea Wahner Hendrickson, Gottfried E. Konecny

**Affiliations:** a Division of Hematology/Oncology, University of California, Los Angeles, CA, United States of America; b David Geffen School of Medicine, University of California, Los Angeles, CA, United States of America; c Department of Oncology, Mayo Clinic, Rochester, MN, United States of America; d Department of Medicine Statistics Core, University of California Los Angeles, Los Angeles, CA, United States of America; e Division of Gynecologic Oncology, University of California Los Angeles, Los Angeles, CA, United States of America

**Keywords:** Trastuzumab Deruxtecan, Antibody-drug conjugates, Ovarian cancer, Endometrial cancer, Real-world outcomes

## Abstract

**Objective**

Trastuzumab deruxtecan (T-DXd), a HER2-directed antibody-drug conjugate, received FDA approval on April 5, 2024 for HER2-expressing solid tumors based on the phase II DESTINY-PanTumor02 trial (*n* = 40 per tumor type). Despite rapid clinical adoption, real-world evidence in gynecologic cancers remains limited. This study aims to address this gap.

**Methods**

This single-center retrospective cohort study included patients with ovarian cancer (OC) or endometrial cancer (EC) who received T-DXd between September 2022 and January 2026. Primary outcomes were real-world progression free survival (rwPFS) and real-world response rate (rwRR). Secondary outcomes included real-world disease control rate (rwDCR), safety, and time on treatment (TOT). Exploratory analysis aimed to evaluate predictors of progression or death on T-DXd using multivariate cox regression.

**Results**

As of February 2026, 40 patients (20 OC, 20 EC) received T-DXd, with a median follow-up of 23 months. All 40 were analyzed for PFS; 37 were response evaluable. HER2 IHC expression was 12.5% 1+, 62.5% 2+, and 20% 3+. Median rwPFS was 6.2 months (95% CI, 3.3–8.6), with a rwRR of 45.9% and rwDCR of 67.6%. rwRR increased significantly with HER2 expression (1+ 16.7%, 2+ 39.1%, 3+ 86%, *p* = 0.03). In multivariate analysis, PARP inhibitor exposure was associated with significantly shorter PFS (HR 4.36, *p* = 0.04). Four patients (10%) developed pneumonitis: three (7.5%) grade 1/2, and one (2.5%) grade 3.

**Conclusions:**

T-DXd demonstrated promising real-world clinical outcomes in OC and EC that mirror DESTINY-PanTumor02. Responses were observed across HER2 IHC expression levels (1+ to 3+), with highest rwRR among HER2 3+. Prior PARP inhibitor exposure was associated with significantly shorter PFS, warranting further exploration.

## Highlights

• We report the real-world outcomes of 40 patients with endometrial or ovarian cancer treated with trastuzumab deruxtecan.
• Real-world median progression-free survival was 6.2 months (95% CI 3.3–8.6) with a real-world response rate (rwRR) of 45.9%.
• Responses were observed across HER2 IHC expression (1+ to 3+), with highest rwRR among patients with HER2 3+ (86%) (*p* = 0.03).
• Prior PARP inhibitor exposure was associated with significantly shorter PFS (HR 4.4, *p* = 0.04) warranting further exploration.

## 1 Introduction

Endometrial and ovarian cancers are the sixth and eighth most common cancers among women worldwide (Endometrial cancer statistics, 2026; Ovarian cancer statistics, 2026). In the advanced or recurrent setting the 5-year survival ranges from 20 to 30%, underscoring the need for effective treatment options (Cancer of the Endometrium, 2026; Cancer of the Ovary, 2025). With the advent of antibody-drug conjugates (ADCs), the recent therapeutic armamentarium has expanded and improved survival outcomes (Silverstein et al., 2025; Drago et al., 2021; Colombo et al., 2024; Moore et al., 2023). ADCs consist of a monoclonal antibody directed against a tumor-associated antigen linked to a cytotoxic payload that induces cell death following internalization into the cancer cell. Two ADCs have received regulatory approval for ovarian and endometrial cancer, mirvetuximab soravtansine (MIRV) for ovarian and trastuzumab deruxtecan (T-DXd) for both ovarian and endometrial, and more than 20 additional ADCs are currently under clinical investigation (Moore et al., 2023; Research C for DE and, 2024).

T-DXd, an ADC targeting HER2 with a topoisomerase-1 inhibitor payload, received FDA approval in April 2024 for HER2-expressing solid tumors (immunohistochemistry [IHC] 3+) based on results from the DESTINY-PanTumor02 trial (Research C for DE and, 2024; Meric-Bernstam et al., 2024). In this multi-cohort basket trial, 40 patients with ovarian and 40 with endometrial cancer achieved objective response rates (ORR) of 45% and 57%, respectively, with the highest responses observed among patients with IHC 3+ tumors (ORR 75% and 84%, respectively) (Meric-Bernstam et al., 2024; Makker et al., 2024). Median progression-free survival (PFS) was 5.9 months for ovarian cancer and 11.1 months for endometrial cancer. Complementing these findings, the STATICE trial evaluated T-DXd in 32 patients with HER2 IHC ≥1+ uterine carcinosarcoma and demonstrated an ORR of 59%. Among the IHC 1+ subgroup (*n* = 10) the ORR was 70%, highlighting that clinical activity may not be restricted to HER2 2+/3+ expression (Nishikawa et al., 2023). Given the impressive response rates in these small trials and significant unmet need in this population, clinical uptake of T-DXd has been rapid. However, real-world data remain limited to small (<10 patient) case series (Andrikopoulou et al., 2024).

To address this gap, we retrospectively evaluated the real-world efficacy and safety of T-DXd in a larger population of patients with ovarian and endometrial cancer.

## 2 Methods

### 2.1 Study design and participants

We conducted a single center, retrospective cohort study of adult patients with ovarian or endometrial cancer treated with T-DXd from September 2022 to January 2026 at the University of California, Los Angeles (UCLA). Patients were identified through the electronic health record (EHR) using International Classification of Diseases (ICD) diagnosis codes and treatment administration records. Inclusion criteria were (1) histologically confirmed ovarian or endometrial cancer; (2) received at least one cycle of T-DXd treatment for their gynecologic cancer. This study was approved by the UCLA Institutional Review Board (IRB-24-1044).

### 2.2 Data collection and variables

**Fig. 1:**
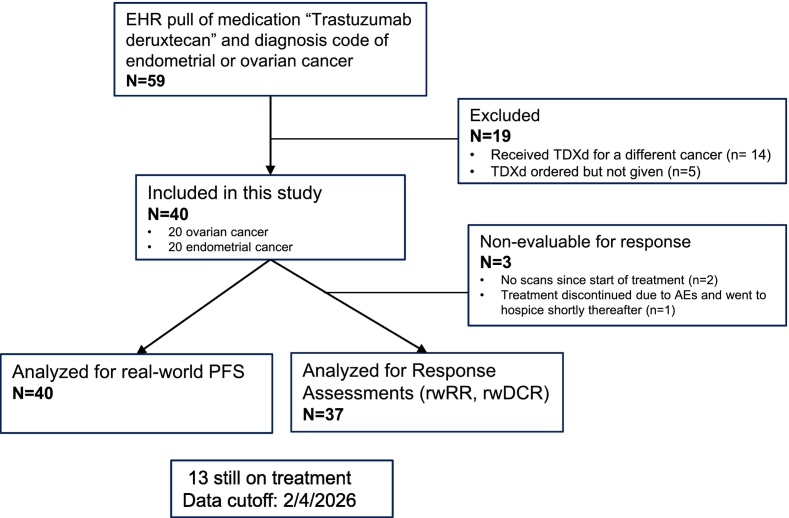
Cohort Diagram *Abbreviations: AE, adverse event; rwDCR, real-worlddisease control rate; rwRR, real-world response rate; PFS, progression-free survival; T-DXd, trastuzumab deruxtecan.*

After identifying 59 patients through EHR extraction, two investigators (O.A. and J.S.) performed manual chart review to confirm eligibility criteria (Fig. 1). Collected variables included demographic and clinical characteristics: age at diagnosis, histologic subtype, endometrial PROMISE molecular classification (Talhouk et al., 2017), prior treatment regimens (including prior exposure to a prior poly (ADP-ribose) polymerase [PARP] inhibitor, topotecan, trastuzumab or antibody-drug conjugate [ADC]), BRCA mutational status, and homologous recombination deficiency (HRD) status.

HER2 immunohistochemistry (IHC) results were recorded for all patients. When multiple HER2 IHC assessments were available, all results were documented, but the highest IHC score was used for analysis mirroring clinical practice. ERBB2 amplification status by next-generation sequencing (NGS) was collected when available.

Treatment-related variables included T-DXd start and end dates, number of treatment cycles, best response per radiology reports, and date of disease progression. Safety endpoints of interest included incidence of grade 1/2 and grade 3/4 adverse events, pneumonitis incidence, and dose reductions.

### 2.3 Outcome variables

The primary outcomes of interest were real-world median progression-free survival (rwPFS) and real-world response rate (rwRR), selected to mirror the key efficacy endpoints of the DESTINY-PanTumor02 trial. Real-world PFS was defined as the time from T-DXd initiation to disease progression (clinical or radiographic) or death from any cause (Mhatre et al., 2023). Patients without a documented rwPFS event were censored at the date they were last known to be alive and progression-free. rwRR was defined by radiology reports as the proportion of patients with best response of real-world complete response (rwCR) or real-world partial response (rwPR) as detailed in Supplementary Tale S1, consistent with prior real-world studies (McKelvey et al., 2024; Ma et al., 2019).

Secondary outcomes of interestincluded real-world disease control rate (DCR), defined as the proportion of patients achieving a best response of rwCR, rwPR, or real-world stable disease (rwSD); time on treatment (TOT); and safety. Exploratory analyses evaluated clinical and molecular predictors of progression or death using cox-proportional hazards ratio. All outcomes were assessed through February 4, 2026 (data-cutoff).

### 2.4 Statistical analysis

All statistical analyses were performed using JMP (version 18; SAS Institute Inc., Cary, NC). Baseline demographic and clinical characteristics and response rates were summarized using descriptive statistics. Survival outcomes (rwPFS) and follow-up times were estimated using the Kaplan-Meier method, with median values and corresponding 95% confidence intervals (CIs) reported. The rwRR (rwCR + rwPR) and its 95% confidence interval were calculated using the Wilson score method. Subgroup comparisons were performed using the log-rank test for PFS and Chi-square or Fisher's exact test for rwRR. As this was a retrospective descriptive analysis, sample size was not predetermined based on statistical power for any single outcome; outcomes are reported descriptively and should be interpreted as hypothesis-generating. Multivariable Cox proportional hazards regression was used to identify predictors of progression; covariates included clinically relevant variables selected a priori. A two-sided *p* value <0.05 was considered statistically significant.

## 3 Results

### 3.1 Baseline characteristics

As of February 2026, we identified 40 patients with ovarian or endometrial cancer that were treated with T-DXd at UCLA and were evaluable for this study. All 40 patients compromised the overall study population analyzed for PFS and safety, however 37 patients were evaluable for response assessment (Fig. 1). Among the 40 patients, 20 had ovarian cancer and 20 had endometrial cancer. Of these, 13 patients received T-DXd prior to its FDA approval in these indications, reflecting off-label, off-study use. Baseline demographic and clinical characteristics are summarized in Table 1. Most patients received T-DXd as monotherapy (*n* = 37, 92.5%); three patients received T-DXd-based combinations, including with bevacizumab (*n* = 1, 2.5%), nivolumab (n = 1, 2.5%), or bevacizumab and pembrolizumab (n = 1, 2.5%).

**Table 1:** Baseline demographics and clinical characteristics by Cancer type.

	Overall Cohort *n* = 40	Ovarian Cancer *n* = 20	Endometrial Cancer n = 20
Median age at T-DXd initiation (range)	69 (37–83)	66.5 (37–76)	70.5 (57–83)
**Histology**			
Serous	26 (65%)	15 (75%)	11 (55%)
Endometrioid	6 (15%)	1 (5%)	5 (25%)
Clear cell	4 (10%)	3 (15%)	1 (5%)
Carcinosarcoma	3 (7.5%)	0	3 (15%)
Poorly differentiated adenocarcinoma	1 (2.5%)	1 (5%)	0
**Endometrial Subtype**			
NSMP	–	–	2 (10%)
p53 abnormal	–	–	17 (85%)
dMMR	–	–	1 (5%)
POLE hypermutated	–	–	0
**Prior lines of therapy**			
Median lines of prior therapy (range)	3 (1−11)	3.5 (1–11)	2 (1–5)
1	8 (20%)	3 (15%)	5 (25%)
2	11 (27.5%)	3 (15%)	8 (40%)
3	9 (22.5%)	4 (20%)	5925%)
4	5 (12.5%)	5 (25%)	0
≥5	7 (17.5%)	5 (25%)	2 (10%)
**BRCA mutational status**			
BRCAmut	4 (10%)	2 (10%)	2 (10%)
BRCAwt	33 (82.5%)	18 (90%)	15 (75%)
Not reported	2 (5%)	0	3 (15%)
**HRD status**			
HRD positive	7 (17.5%)	5 (25%)	2 (10%)
HRD negative	14 (35%)	10 (50%)	4 (20%)
Not reported	19 (47.5%)	5 (25%)	14 (70%)
**Prior lines of treatment**			
Prior ADC	9 (22.5%)	7 (35%)	2 (10%)
Prior MIRV	7 (17.5%)	7 (35%)	0
Other ADC	3 (7.5%)	1 (5%)⁎⁎⁎	2 (10%)
Prior PARP inhibitor	8 (20%)	7 (35%)	1 (5%)
Prior trastuzumab	5 (12.5%)	3 (15%)	2 (10%)
Prior topotecan	3 (7.5%)	3 (15%)	0
**HER2 expression**⁎			
IHC 1+	6 (15%)	5 (25%)	1 (5%)
IHC 2+	25 (62.5%)	10 (50%)	15 (75%)
IHC 3+	8 (20%)	4 (20%)	4 (20%)
No IHC reported⁎⁎	1 (2.5%)	1 (5%)	0
ERBB2 amp	14 (35%)	8 (40%)	6 (30%)

Abbreviations: dMMR, deficient mismatch repair; EC, endometrial cancer; ER+, estrogen receptor–positive; HGSOC, high-grade serous ovarian cancer; LGSOC, low-grade serous ovarian cancer; NSMP, no specific molecular profile; POLE, DNA polymerase epsilon hypermutated; PROMISE, Proactive Molecular Risk Classifier for Endometrial Cancer; TCGA, The Cancer Genome Atlas.

⁎ if there were multiple HER2 IHC results we reported the highest HER2 result.

⁎⁎ 1 patient without HER2 IHC testing had ERBB2 copy number gain (CNG) by NGS testing.

⁎⁎⁎ One patient received both MIRV and an ADC on a clinical trial prior to TDXd.

### 3.2 Efficacy analysis of the overall cohort

Among the entire cohort, the median PFS was 6.2 months (95% confidence interval [CI] 4.1–8.6) with a rwRR of 45.9% (17/37; 95% CI: 30.8–61.1) and rwDCR of 67.6% (25/37; 95% CI 51.1–81.4) (Table 2). Best responses included 5/37 (13.5%) complete responses, 12/37 (32.4%) partial responses, 8/37 (21.6%) stable disease and 12/37 (32.4%) progressive disease. The median follow-up time was 23 months. The median duration of treatment was 6.2 months (95% CI 2.7–7.6) with a median of 6 cycles administered (range, 1–31). The 6 month PFS was 50.2% (95% CI: 33.4%–66.6%) and the 12-month PFS was 22.9% (95% CI, 7.7%–38.2%)..

**Table 2:** Progression free survival and response by cancer type.

	Overall Cohort n = 40	Ovarian n = 20	Endometrial n = 20
Median PFS, mos (95% CI)	6.2 (3.3–8.6)	5.8 (3.3–11.3)	6.5 (1.4–8.5)
***Response-evaluable, n***	37	19	18
rwRR (95% CI)	45.9% (30.8–61.1)	47.4% (27.3–68.3)	44.4% (24.6–66.3)
rwDCR (95% CI)	67.6% (51.1–81.4)	73.7 (51.2–88.2)	61.1% (38.6–79.7)
rwCR	5 (13.5%)	2 (10.5%)	3 (16.7%)
rwPR	12 (32.4%)	7 (36.8%)	5 (27.8%)
rwSD	8 (21.6%)	5 (26.3%)	3 (16.7%)
rwPD	12 (32.4%)	5 (26.3%)	7 (38.9%)

Abbreviations: CI, confidence interval; mos, months; PFS, progression-free survival; rwCR, real-world complete response; rwDCR, real-world disease control rate; rwPD, real-world progressive disease; rwPR, real-world partial response; rwRR, real-world response rate; rwSD, real-world stable disease.

**Fig. 2:**
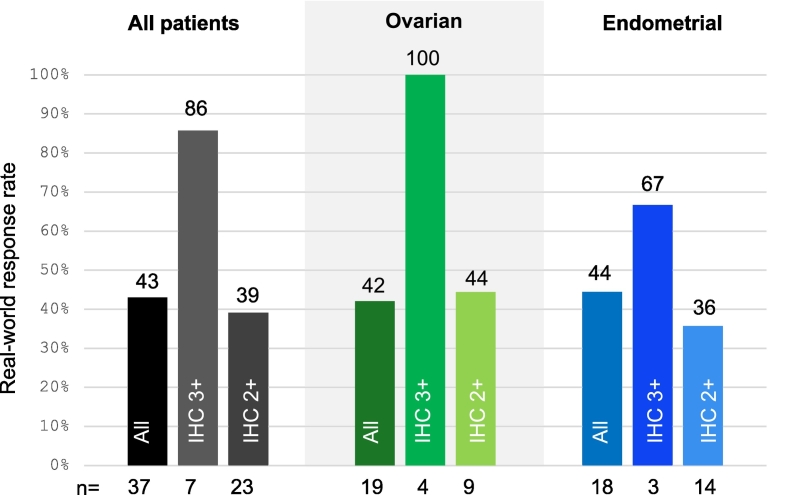
Real-world Response Rates by HER2 IHC status and Cancer Type.

Within the cohort, 15% (6/40) had HER2 IHC 1+, 62.5% (25/40) had HER2 IHC 2+, and 20% (8/40) had HER2 IHC 3+ expression levels. Median PFS was similar across HER2 subgroups (1+: 6.1 months [95% CI 1.1–26.6], 2+: 6.0 months [1.7–8.5], 3+: 6.4 months [3.0-NR]; *p* = 0.61). However, rwRR increased significantly with higher HER2 expression (1+: 16.7%, 2+: 39.1%, 3+: 85.7%, *p* = 0.03) (Fig. 2).

### 3.3 Ovarian cancer

**Fig. 3:**
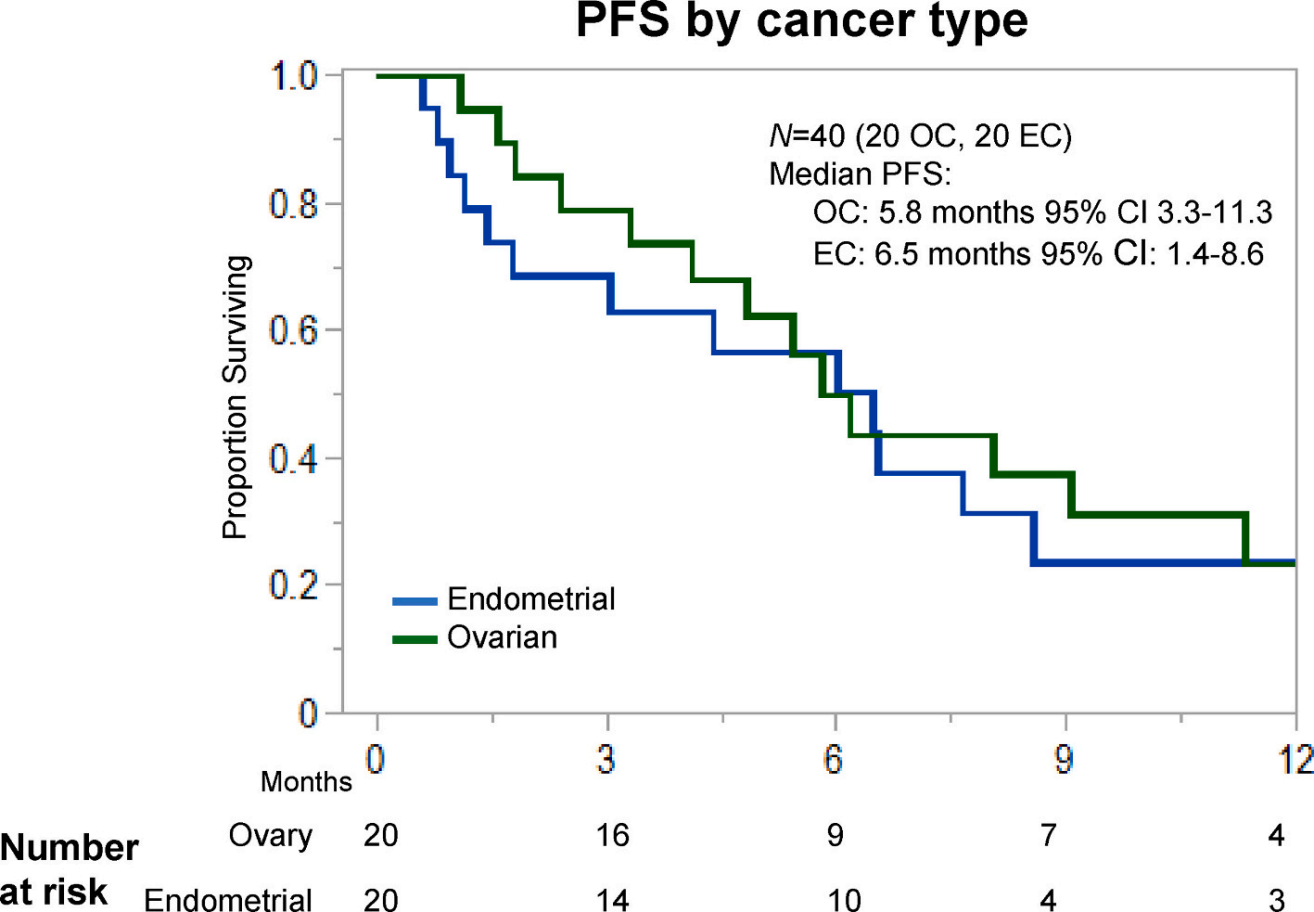
Progression Free Survival by Cancer Type. *Abbreviations: EC, endometrial cancer; OC; ovarian cancer.*

**Fig. 4:**
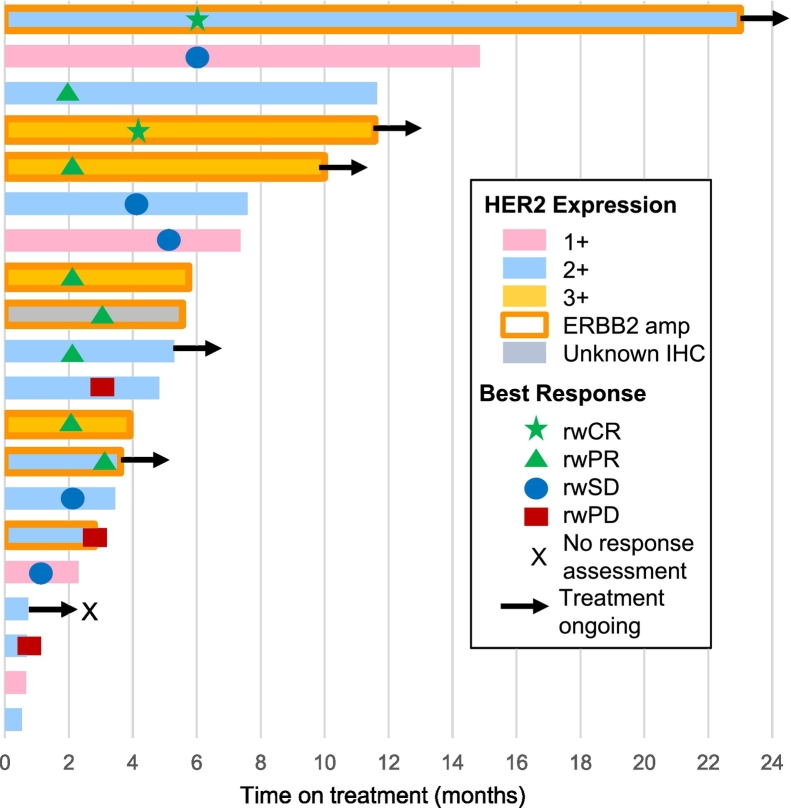

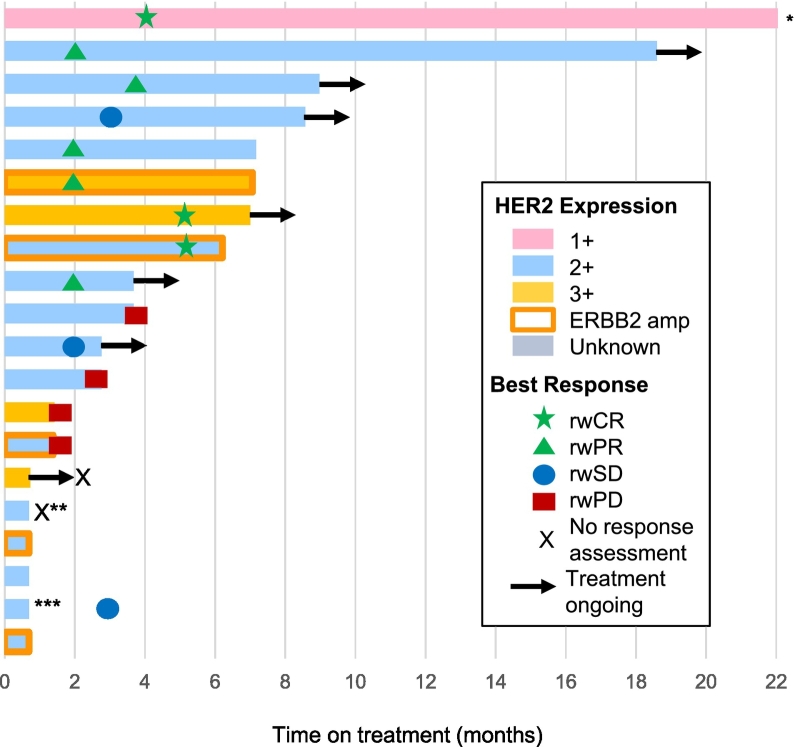
A. Time on treatment for patients with ovarian cancer by HER2 IHC status. B. Time on treatment for patients with endometrial cancer by HER2 IHC status *Stopped due to insurance issues, and was on surveillance for another year; **Got 1 dose had nausea and decided to go on hospice; ***Neutropenic fever and sepsis after the first dose of T-DXd but had stable disease on subsequent scan. Abbreviations: ADC, antibody–drug conjugate; amp, amplification; IHC, immunohistochemistry; HER2, human epidermal growth factor receptor 2; PARP, poly(ADP-ribose) polymerase; rwCR, real-world complete response; rwPD, real-world progressive disease; rwPR, real-world partial response; rwSD, real-world stable disease.

Among 20 patients with ovarian cancer, the median PFS was 5.8 months (3.3–11.3), rwRR was 47.4% (9/19; 95% CI: 27.3–68.3) and rwDCR was 73.7 (14/19; 95% CI: 51.2–88.2) (Table 2**,** Fig. 3). Best responses were 2/19 (10.5%) complete responses, 7/19 (36.8%) partial responses, 3/19 (15.8%) stable disease, and 5/19 (25%) progressive disease. Fig. 4. A shows time on treatment by IHC status, with the longest patient on treatment for 23 months and ongoing at the time of data-cutoff. Among the patients with ovarian cancer, 70% had received 3 or more lines of prior therapy including 7/20 (35%) patients with prior MIRV. Of the patients with prior MIRV, the rwRR was 14.2% (95% CI: 2.5–51.3) and median PFS was 8.0 months (1.5–15.8). Of the three patients that had received topotecan the rwRR was 0% and the median PFS was 3.3 months (1.1–8.0). Of the three patients that had received trastuzumab, the rwRR was 66% and median PFS was 4.8 months (3.3-NR).

### 3.4 Endometrial cancer

Among 20 patients with endometrial cancer (18 response-evaluable), the median PFS was 6.5 months (95% CI: 1.4–8.5), rwRR was 44.4% (8/18; 95% CI: 24.5–66.2) and rwDCR was 61.1% (11/18; 95% CI: 38.6–79.7) (Table 2**,** Fig. 3). Best responses were 3/18 (16.7%) complete responses, 5/18 (27.7%) partial responses, 3/18 (16.7%) stable disease and 7/18 (38.8%) with progressive disease. Fig. 4. AB shows time on treatment by HER2 IHC status, with the longest patient on treatment for 22.5 months. Most patients with endometrial cancer (16/20, 80.0%) had received prior immunotherapy; in these individuals, the rwRR was 40.0% and the median PFS was 6.0 months (95% CI: 1.1–8.6). No patients with endometrial cancer were treated with topotecan. Of the two patients with endometrial cancer who had prior trastuzumab the rwRR was 0%. The median number of prior lines of therapy was 2 (range: 1–5); 13 patients (65.0%) had received ≤2 prior lines, while 7 patients (35.0%) had received >2 prior lines. The median PFS for patients with ≤2 prior lines was 6.5 months (95% CI: 0.8–26.6) compared to 3.0 months (95% CI: 0.9–8.6) for those with >2 prior lines (*p* = 0.33).

### 3.5 HER2 concordance

Fifteen patients had multiple HER2 IHC assessments available for comparison: 14 patients had biopsies from different time points, and one patient had concurrent biopsies from two separate sites. Among these, three patients had HER2 IHC performed at three different time points.

The overall discordance rate between HER2 IHC assessments was 86.7% (13/15 patients) (Supplemental Fig. S1). Among discordant cases, 60% demonstrated lower HER2 expression on repeat testing and 26% showed higher expression. Notably, 80% of patients (*n* = 12) had discordant results that would alter treatment eligibility (transition between 0/1+ and 2+/3+ categories).

### 3.6 Predictors of response

In multivariable cox proportional regression analysis, accounting for age at T-DXd initiation, cancer type, HER2 IHC score, prior topotecan, number of lines of therapy prior to TDXd, prior PARP inhibitor, and prior ADC treatment, only prior PARP inhibitor was associated with a shorter PFS (Hazard ratio: 4.36, 95% CI 1.0–18.4, *p* = 0.04, **Supplemental Table S2**). The median PFS of patients with prior PARP inhibitors was 3.7 months (95% CI 0.9–9.0) compared to 6.5 months (4.4–11.3) without prior PARP inhibitors (*p* = 0.03) (Supplemental Fig. S2). When limiting to patients with only ovarian cancer, the median PFS of patients with prior PARP was 4.1 mos (1.1–8.0) compared to 9.1 mos (1.8-NR), *p* = 0.02. Among the homologous recombination proficient patients with ovarian cancer (*n* = 15), three were treated with a PARP and the median PFS of PARP-treated patients in this subgroup was 4.8 mos (2.4-NR) compared to 11.3 (1.8-NR) in PARP-naïve patients. Median PFS among BRCA-mutated patients (*n* = 4) was 1.0 month (95% CI 0.59–3.3) compared with 6.5 months (95% CI 4.8–9.1) among BRCA wild-type patients (*p* < 0.001). Among the 21 patients with HRD testing available, median PFS was 3.3 months (95% CI 0.59–8.0) in HRD-positive patients and 11.3 months (95% CI 4.8–26.6) in HRD-negative patients (*p* = 0.005).

### 3.7 Safety

The most common adverse events were nausea (67.5%), anemia (72.5%), neutropenia (50.0%), constipation (42.5%), and white blood cell (WBC) decrease (40.0%), with most events being Grade 1/2 in severity **(Supplemental Table S3**). Grade 3/4 adverse events were primarily hematologic and included anemia (25.0%), neutrophil decrease (17.5%), WBC decrease (7.5%), and thrombocytopenia (5.0%), while Grade 3/4 nonhematologic toxicities were uncommon, with only isolated cases of nausea, vomiting, and neuropathy (2.5% each).

Four patients (10%) developed pneumonitis: two grade 1, one grade 2, and one grade 3. Detailed explanations of their clinical course can be found in **Supplemental Table S4**. The patient who developed grade 3 pneumonitis had pre-existing treatment-related pneumonitis (from pegylated liposomal doxorubicin and/or MIRV) at the time of T-DXd initiation. On retrospective review of imaging reports, six additional patients had incidental findings concerning for an infectious or inflammatory pulmonary process of unclear significance that had not been documented by the treating physician and were not accompanied by patient-reported symptoms.

Dose reductions occurred in 27.5% of patients, on average after 4 cycles. Three patients initiated treatment at reduced doses (two at 4.4 mg/kg, one at 3.2 mg/kg). Indications for initial dose reduction included fatigue (*n* = 2), neuropathy (n = 2), thrombocytopenia (n = 2), neutropenia (*n* = 1), anemia (n = 1), and weight loss (n = 1). Four patients (10%) underwent a second dose reduction for fatigue (n = 2), neuropathy (n = 2), and intermittent dyspnea (n = 1), with one patient ultimately reduced to 2.2 mg/kg for ongoing neuropathy. rwRR did not differ significantly between patients who underwent dose reduction and those who did not (46.1% vs 45.4%, *p* = 0.86).

## 4 Discussion

This study describes a real-world cohort of patients with HER2-expressing endometrial and ovarian cancer treated with T-DXd. In this heavily pre-treated population, T-DXd demonstrated meaningful clinical activity (median PFS of 6.2 months and rwRR of 46%), with the highest response rate in the HER2 3+ subgroup (rwRR 86%). Clinical benefit was evident across all HER2 expression levels, including HER2-low disease.

A key strength of this study is addressing a critical gap in our understanding of T-DXd's real-world efficacy. The FDA approval of T-DXd for HER2-expressing gynecologic cancers was based solely on DESTINY-PanTumor02, which enrolled only 40 patients per tumor type. Despite this limited evidence base, broad clinical adoption has followed. In this interval before larger trials mature, real-world studies are essential to validate efficacy and characterize toxicity across a broader, more representative patient population. Our findings closely corroborate DESTINY-PanTumor02, with a rwRR of 43% in the overall population and 86% in the HER2 3+ subgroup, compared to 51% and 75% in DestinyPanTumor-02, respectively (Meric-Bernstam et al., 2024). Median PFS in our ovarian cancer cohort was 5.8 months, nearly identical to the 5.9 months reported in DESTINY-PanTumor02. The published real-world series to date includes ten patients from a single institution, reporting an ORR of 50% (80% in HER2 3+ expressors) and median PFS of 5.4 months (Andrikopoulou et al., 2024). A recent abstract presented at the annual Society of Gynecologic Oncology 2026, evaluated 75 patients at a single academic institutions with gynecologic cancers treated with TDXd and found a similar median PFS of 6.3 months (Flint et al., n.d.). Beyond this, real-world evidence remains limited to case reports (Rose, 2023; Kong et al., 2024; Xing et al., 2024). As utilization expands, our cohort represents a meaningful addition to this evidence base, and multi-institutional real-world data collection and larger controlled clinical trials will be essential.

The most notable difference in our study compared to DESTINY-PanTumor02 was the real-world PFS in our endometrial cancer cohort. Our cohort of patients with EC had a median PFS of 6.5 months, compared to 11.1 months in DESTINY-PanTumor02, and the HER2 3+ subgroup had a median PFS of 6.4 months versus 28 months. This is likely driven in part by the small size of our HER2 3+ endometrial cohort (*n* = 4), two of whom had lower HER2 expression on more recent biopsies, which may underestimate true efficacy in this subgroup. Additionally, inherent differences between trial and real-world populations, including patients that are more heavily pretreated, with comorbidities and lower performance status typically excluded from trials, likely contribute to the differences observed and underscore the complementary value of both data sources (Wilson and Booth, 2024).

In our cohort, higher HER2 expression was associated with a significantly higher rwRR to T-DXd, a finding consistent with data from DESTINY-PanTumor02 and mechanistically supported by the dependence of ADC uptake on target density. Notably, however, breast cancer trials of T-DXd in HER2-low (IHC 1+/2+ ISH−) and HER2-ultralow (IHC 0 with faint membrane staining) disease have demonstrated ORRs of 56.5–61.8%, comparable to those observed in HER2-high expressors (∼61%) (Modi et al., 2022; Bardia et al., 2024; Modi et al., 2020). In our cohort, despite differences in rwRR across HER2 expression levels, median PFS did not differ significantly, with the HER2 1+ cohort still achieving a median PFS of 6 months. This may suggest meaningful T-DXd activity even in lower HER2-expressing gynecologic cancers, though this warrants prospective investigation. The impact of HER2 temporal and spatial heterogeneity could also play a role in responses seen at lower HER2 levels. Among the 15 patients with multiple HER2 IHC assessments available, the discordance rate was 87%, with 80% of discordant cases representing a clinically meaningful shift between the 0/1+ and 2+/3+ categories that would alter treatment eligibility. This is consistent with another study reporting 68% discordance among 19 paired ovarian cancer biopsies, and further compounded by inter-pathologist discordance of 22% on the same biopsy (Kim et al., 2024; Salinaro et al., 2025). Collectively, these findings underscore the lack of precision in HER2 testing, the need for standardized HER2 testing protocols in gynecologic cancers, and raise the possibility that HER2 “low”, IHC 0/1+, disease may benefit from T-DXd.

Beyond the primary efficacy findings, an intriguing exploratory observation was the association between prior PARP inhibitor exposure and significantly worse PFS (3.7 vs. 6.5 months, HR 4.7). While exploratory, this is biologically plausible. PARP inhibitors are well established to induce subsequent chemoresistance through multiple mechanisms: restoration of homologous recombination repair, upregulation of multidrug efflux pumps, replication fork stabilization, and alternative pathway activation (Chiappa et al., 2021; Lukashchuk, 2026; Washington and Moore, 2022). Multiple retrospective studies demonstrate PARP inhibitor exposure compromises subsequent platinum response (Frenel et al., 2022; Vacheresse, 2026; Park et al., 2022). Whether this extends to ADC therapies has not previously been reported. Notably, T-DXd's topoisomerase I inhibitor payload induces DNA single-strand breaks that require functional PARP1 for repair, and emerging evidence implicates efflux pump upregulation in T-DXd resistance specifically — suggesting at least two plausible mechanisms by which prior PARP exposure could attenuate T-DXd activity (Lee et al., 2024; Sledge et al., 2025). Prospective studies are needed to validate this signal and evaluate whether combination strategies can overcome PARP-induced resistance in this setting.

Alongside these efficacy findings, the safety profile of T-DXd warrants attention. In our cohort, 10% developed physician-diagnosed pneumonitis, consistent with reported real-world rates of 9–22% and a pooled trial incidence of 12.4% (Liao et al., 2025; Azhar et al., 2025; *Pulmonary toxicities in patients (pts) with metastatic breast cancer (mBC) treated with trastuzumab deruxtecan (T-DXd): The Mayo Clinic experience*, 2026; Kawakami et al., 2025). Two of the four patients with pneumonitis in our cohort had pre-existing lung inflammation — a population typically excluded from trials — suggesting that real-world use may expose a higher-risk group. Other real-world studies suggest baseline interstitial lung abnormalities and pulmonary comorbidities may be predictors of pneumonitis (Azhar et al., 2025; *Pulmonary toxicities in patients (pts) with metastatic breast cancer (mBC) treated with trastuzumab deruxtecan (T-DXd): The Mayo Clinic experience*, 2026; Kawakami et al., 2025). An important concern is the potential for underdiagnosis: in our cohort, several patients had possible inflammatory CT findings not formally attributed to pneumonitis by their treating physicians. A real-world study of 68 patients with breast cancer had a radiologist evaluate the CT scans throughout T-DXd treatment. By treating physician assessment, 7.4% developed grade 1 pneumonitis and 4.4% developed grade 2 pneumonitis. However, by radiologist review 23.5% had grade 1 pneumonitis and 4.4% grade 2 pneumonitis (*Association of pretreatment chest CT abnormalities with trastuzumab deruxtecan–associated pneumonitis*, 2026). This discordance has real clinical stakes — interstitial lung disease can be fatal, and prompt recognition, discontinuation of T-DXd, and initiation of corticosteroids are essential. As T-DXd use expands into real-world gynecologic oncology populations with greater comorbidity burden, systematic radiologic surveillance and heightened clinical vigilance will be critical to mitigating this risk.

Limitations of this study include its single-center design, which may limit generalizability, and the absence of a comparator arm. The heterogeneity of the patient population resulted in small sample sizes within individual tumor subtypes. Since these are real-world patients the rwRR was obtained by radiology report which may not reflect the same stringent trial endpoints and therefore potentially lead to an overestimation of the ORR in our cohort. Three patients received T-DXd-based combination therapy (*n* = 1 bevacizumab, n = 1 nivolumab, n = 1 bevacizumab/pembrolizumab); as such, the reported efficacy outcomes for these patients cannot be attributed to T-DXd alone and may overestimate the activity of T-DXd monotherapy in this cohort. Given the small sample size of the overall cohort, we elected to include these patients in the primary analysis, as we expect the contribution of the combination partners to be small relative to T-DXd. To assess the potential impact of their inclusion, we conducted a sensitivity analysis excluding these three patients (*n* = 34 response-evaluable) and found a rwRR of 50.0% and median PFS of 6.0 months (95% CI 4.1–8.0), nearly identical to the full-cohort estimates of 45.9% (95% CI 30.8–61.1) and 6.2 months, respectively. The heterogeneity of tumor biopsies and lack of standardization of testing make interpretation of HER2-IHC status in relation to PFS harder to interpret. Given the small sample size of patients who had PARP exposure (*n* = 8) this finding is strictly hypothesis generating.

## 5 Conclusion

This real-world cohort of patients with HER2-expressing endometrial and ovarian cancer treated with T-DXd demonstrated meaningful clinical activity with an ORR of 43% and median PFS of 6.2 months. Response rates were highest in the HER2 3+ subgroup, though clinical benefit was observed across all HER2 expression levels. The safety profile was consistent with known T-DXd toxicity, with pneumonitis representing a critical toxicity requiring heightened vigilance, particularly in real-world populations with greater comorbidity burden. The association of prior PARP inhibitor exposure and T-DXd efficacy warrants further investigation. Multi-institutional real-world studies and prospective clinical trials are essential to fully define the efficacy and safety profile of T-DXd in gynecologic cancers.

## Disclosures

During the preparation of this work the authors utilized Claude in order to improve readability and language. After using this tool/service, the authors reviewed and edited the content as needed and take full responsibility for the content of the publication.

## CRediT authorship contribution statement

**Jordyn Silverstein:** Writing – review & editing, Writing – original draft, Visualization, Validation, Supervision, Project administration, Methodology, Investigation, Formal analysis, Data curation, Conceptualization. **Oladunni Alomaja:** Writing – review & editing, Formal analysis, Data curation. **Deandra Chatram:** Writing – review & editing, Formal analysis, Conceptualization. **Maryam Hajiabbasi:** Writing – review & editing, Investigation, Formal analysis, Data curation. **Eliya Shachar:** Writing – review & editing, Conceptualization. **Chi-Hong Tseng:** Writing – review & editing, Software, Methodology, Formal analysis, Conceptualization. **Shivani Thaker:** Writing – review & editing, Conceptualization. **Aditya Bardia:** Writing – review & editing, Supervision, Conceptualization. **Beth Karlan:** Writing – review & editing, Supervision. **Andrea Wahner Hendrickson:** Writing – review & editing, Supervision, Formal analysis, Data curation. **Gottfried E. Konecny:** Writing – review & editing, Writing – original draft, Supervision, Methodology, Funding acquisition, Data curation, Conceptualization.

## Declaration of competing interest

The authors declare that they have no known competing financial interests or personal relationships that could have appeared to influence the work reported in this paper.
