# Supplementary material for: Real-world clinical outcomes of Trastuzumab Deruxtecan in HER2-expressing ovarian and endometrial Cancer
Source: Gynecol Oncol Rep. 2026 Aug 12;66:102183. doi: 10.1016/j.gore.2026.102183 (PMC13487597; doi:10.1016/j.gore.2026.102183)
Supplement: Supplementary file 1 [file mmc3.docx]

Supplementary Tables

**Supplemental Table S1.** Definition of real-world response rates^16^

**Supplemental Table S2.** Multivariate Cox Proportional Hazards model to predict progression or death from Trastuzumab Deruxtecan

**Supplemental Table S3.** Treatment-related adverse events with trastuzumab deruxtecan

**Supplemental Table S4.** Detailed clinical course of patients with pneumonitis

**Supplemental Table S1.**

| **Rw-Response Category** | **Clinician Assessment** | **Terms to Map to Categories** |
| --- | --- | --- |
| **Real-world complete response (rwCR)** | Complete resolution of disease, no evidence of disease | - Complete response, Full/Complete resolution - Remission, Complete remission - All lesions have disappeared, All lesions resolved - No evidence of disease, NED - No disease present, No sites of disease |
| **Real-world partial response (rwPR)** | Partial reduction in disease burden (decrease in size or extent), with disease still present | - Improved disease - Responding disease - Partial response, Partial remission, PR - Positive, significant, marked, good, meaningful, substantial, vast, excellent, near complete |
| **Real-world stable disease (rwSD)** | No change in disease burden (in extent or severity) noted. | - Disease is stable, Stable disease, SD - Not progressed or not improved (Absence of positive/significant/marked change) - No change in disease burden - Slight (slight or minimal) response or improvement or worsening noted in conjunction with notes of stable disease   *Note: a clinician may note stable disease in reference to the last assessment, rather than in comparison to the baseline tumor burden. Therefore, rwSD after an assessment of rwPR or rwCR will not be considered progressive disease. |
| **Real-world progressive disease (rwPD)** | Increase in disease and/or presence of new lesions | - Progressed, Progression, PD - Worsening of disease - Indication of new metastasis, new lesions |
| **Real-world mixed response (rwMR)** | Combination of improved and worsened disease | - Mixed response - Noted terms from rwCR/rwPR and rwPD categories |
| **Not evaluable (NE)** | Response is not evaluable (clinician noted response was not evaluable, other) | - Not evaluable - Indeterminate - Inconclusive to provider or in the data capture - Pseudoprogression |

**Supplemental Table S2.**

| **Variable** | **Category** | **Hazards ratio** | **95% Confidence Interval** | **p-value** |
| --- | --- | --- | --- | --- |
| Age at T-DXd initiation | - | 0.97 | 0.91-1.03 | 0.33 |
| HER2 IHC (ref 1+) | 2+ | 2.13 | 0.63-7.19 | 0.46 |
|  | 3+ | 1.74 | 0.32-9.39 |  |
| Prior PARP (ref: no) | yes | 4.36 | 1.03-18.4 | **0.04** |
| Prior Topotecan (ref: no) | yes | 0.29 | 0.03-3.24 | 0.32 |
| Prior ADC (ref: no) | yes | 0.65 | 0.18-2.31 | 0.51 |
| Lines of treatment prior to T-DXd | - | 1.29 | 1.82-0.77 | 0.14 |
| Cancer type (ref: endometrial) | ovarian | 0.45 | 0.16-1.31 | 0.14 |

**Supplemental Table S3.**

| **Adverse Events (n=40)** | **Total** | **Grade 1/2 n (%)** | **Grade 3/4 n (%)** |
| --- | --- | --- | --- |
| Any adverse event | 38 (95.0%) | 37 (92.5%) | 18 (45.0%) |
| WBC decreased | 16 (40.0%) | 13 (32.5%) | 3 (7.5%) |
| Neutrophil decreased | 20 (50.0%) | 13 (32.5%) | 7 (17.5%) |
| Anemia | 29 (72.5%) | 19 (47.5%) | 10 (25.0%) |
| Platelet count decreased | 11 (27.5%) | 9 (22.5%) | 2 (5.0%) |
| AST increased | 10 (25.0%) | 10 (25.0%) | 0 |
| ALT increased | 2 (5.0%) | 2 (5.0%) | 0 |
| Nausea | 27 (67.5%) | 26 (65.0%) | 1 (2.5%) |
| Constipation | 17 (42.5%) | 17 (42.5%) | 0 |
| Vomiting | 14 (35.0%) | 13 (32.5%) | 1 (2.5%) |
| Diarrhea | 14 (35.0%) | 14 (35.0%) | 0 |
| Neuropathy | 12 (30.0%) | 11 (27.5%) | 1 (2.5%) |
| Pneumonitis | 4 (10.0%) | 3 (7.5%) | 1 (2.5%) |

**Supplemental Table S4.**

| **Patient ID** | **Pneumonitis max grade** | **Cycle onset** | **Clinical Course** |
| --- | --- | --- | --- |
| 6 | grade 3 | 2 | Prior to initiation of TDXd, the patient had imaging findings concerning for pneumonitis related to prior treatment with pegylated liposomal doxorubicin and/or mirvetuximab soravtansine. Shortly after the second cycle of T-DXd, she developed dyspnea and was admitted to the hospital requiring supplemental oxygen. CT imaging demonstrated multifocal pulmonary parenchymal consolidations and ill-defined nodules, most prominent in the right lower lobe, concerning for lymphangitic carcinomatosis with possible superimposed pneumonia. She was treated with antibiotics, and corticosteroids were deferred. Her oxygenation improved with antibiotic therapy. However, follow-up CT imaging after discontinuation of T-DXd demonstrated worsening lower lung subpleural and peribronchovascular consolidative opacities, raising concern for organizing pneumonia in the setting of drug toxicity. |
| 14 | grade 2 | 16 | At the time of symptom onset (dyspnea) she was on both T-DXd and Nivolumab and imaging showed new ground glass opacities. Treatment was held, and steroids and antibiotics were initiated. Follow-up imaging demonstrated stability with symptomatic improvement; however, the patient subsequently progressed and T-DXd was discontinued. |
| 36 | grade 1 | 10 | Patient was asymptomatic and her routine CT imaging showing new patchy airspace opacities in the right lung concerning for inflammatory in etiology. She was treated with antibiotics and provided steroids for home use if symptoms developed. Her imaging findings eventually resolved while continuing therapy. |
| 41 | grade 1 | 6 | Patient had a history of cryptogenic organizing pneumonia and developed imaging findings suggestive of an infectious or inflammatory process while on T-DXd. T-DXd was held pending workup, and findings improved without requiring steroids. Treatment was not restarted; the patient eventually progressed and was initiated on next line of therapy. |
